# Hair Cortisol Concentrations Are Associated with Dental Anxiety during Pregnancy

**DOI:** 10.3390/dj9040042

**Published:** 2021-04-11

**Authors:** Hilja Viitaniemi, Auli Suominen, Linnea Karlsson, Paula Mustonen, Susanna Kortesluoma, Kari Rantavuori, Ana João Rodrigues, Bárbara Coimbra, Hasse Karlsson, Satu Lahti

**Affiliations:** 1 Department of Community Dentistry, University of Turku, 20014 Turku, Finland; hilja.s.viitaniemi@utu.fi (H.V.); auli.suominen@utu.fi (A.S.); 2 FinnBrain Birth Cohort Study, Department of Clinical Medicine, Turku Brain and Mind Center, University of Turku, 20014 Turku, Finland; linnea.karlsson@utu.fi (L.K.); pmmust@utu.fi (P.M.); sukort@utu.fi (S.K.); kari.rantavuori@utu.fi (K.R.); hasse.karlsson@utu.fi (H.K.); 3 Centre for Population Health Research, University of Turku and Turku University Hospital, 20014 Turku, Finland; 4 Department of Psychiatry, University of Turku and Turku University Hospital, 20014 Turku, Finland; 5 Department of Child Psychiatry, University of Turku and Turku University Hospital, 20014 Turku, Finland; 6 Department of Pediatric Dentistry and Orthodontics, University of Turku, 20014 Turku, Finland; 7 Life and Health Sciences Research Institute (ICVS), School of Medicine, University of Minho, 4710-057 Braga, Portugal; ajrodrigues@med.uminho.pt (A.J.R.); barbaracoimbra@med.uminho.pt (B.C.)

**Keywords:** dental anxiety, hair cortisol, depression, general anxiety, FinnBrain

## Abstract

Dental anxiety (DA) and hair cortisol concentrations (HCC) are associated with psychological symptoms and vary during pregnancy. We aimed to examine the association between HCC and DA at two points of pregnancy. Participants were pregnant mothers (n = 533) drawn from the FinnBrain Birth Cohort Study donating a hair sample at gestational week (gwk) 24 (n = 442) and/or at delivery (n = 176) and completed questionnaires on DA. Two groups, HCC1 and HCC2, treated as separate in the analyses, were formed according to the hair sample donation time i.e., gwk24 and delivery. 85 subjects were included in both groups. MDAS, EPDS, and SCL-90 were used to measure DA, depressive and anxiety symptoms, respectively, at gwk14 for the HCC1 group and gwk34 for the HCC2 group. The association between DA and HCC was studied with a binary logistic regression model, adjusted for anxiety and depressive symptoms, age, BMI, and smoking status. Individuals with high DA had lower HCC levels at gwk24 (OR = 0.548; 95% CI = 0.35–0.86; *p* = 0.009), but the association was not statistically significant at the delivery (OR = 0.611; 95% CI = 0.28–1.33; *p* = 0.216). The independent association between HCC and DA in pregnant women suggests that long-term cortisol levels could play a role in the endogenous etiology of DA. Further studies are however, needed.

## 1. Introduction

Dental anxiety is a prevalent problem that often leads to avoidance or symptom-driven use of dental care, especially among those with high dental anxiety [1,2,3]. Dental anxiety and avoidance can consequently lead to poor perceived and clinically determined oral health [4,5]. Besides exogenous sources (such as direct and indirect experiences), dental anxiety can originate from endogenous sources [6]. These internal etiological factors have been referred as a ‘constitutional vulnerability to (dental) anxiety disorders’ [7]. Dental anxiety has been associated with several psychological disorders and symptoms, such as phobias, depression, and general anxiety [8,9,10,11,12,13,14,15,16,17,18,19,20], supporting the common constitutional vulnerability and endogenous etiology. Of the two aspects of dental anxiety, anticipatory dental anxiety has shown a stronger association with anxiety and depression than treatment-related dental anxiety [20,21]. This supports the endogenous etiology of dental anxiety. During pregnancy and after delivery, dental anxiety changed differently in fathers and mothers expecting a baby [17,22]. In addition, the association of the changes between dental anxiety, general anxiety, and depressive symptoms (changes during pregnancy not significant for the latter two in this population) were different among fathers and mothers [23], suggesting that hormonal changes may also play a role in these changes.

One of the hormonal changes during pregnancy relates to cortisol levels that tend to increase up to 2–3 fold towards the end of pregnancy via a complex feed-forward system between cortisol placental corticotropin-releasing hormone (CRH) secretion [24]. This leads to the attenuated reactivity of the maternal hypothalamic-pituitary-adrenal (HPA) axis towards the end of the pregnancy.

Hair cortisol concentration (HCC) is a retrospective measure of long-term cortisol secretion [25] that has been associated with psychological symptoms to varying degrees. A systematic review by Koumantarou Malisiova et al. [26] revealed inconsistent associations between HCC and anxiety disorders, whereas affective disorders, such as depression, were associated with elevated HCC in the majority of the studies included. The diverging results can be partially explained by mental disorder comorbidities of the participants and the heterogeneity of the disorders. The association is often studied with self-report questionnaires of perceived stress and symptoms. The inconsistency of the results may also be due to methodological aspects regarding the HCC analysis [26,27,28].

Maternal psychological distress has been shown to be linked with cortisol levels [29,30]. In studies performed during pregnancy, both a positive association and no association were found between HCC and prenatal psychological distress in a systematic review [31]. A recent study by Mustonen et al. [32] examined association between prenatal HCC levels and psychological distress symptom trajectories during pregnancy. They reported a negative association between pregnancy-related anxiety and HCC, and elevated HCC in participants experiencing continuously high levels of depressive symptoms during pregnancy.

These studies suggest that psychological symptoms and disorders are associated with alterations in HCC levels which vary during pregnancy. Similarly, dental anxiety and its association with general anxiety and depressive symptoms vary during pregnancy suggesting that long-term cortisol levels could be associated with dental anxiety. However, according to our knowledge, there are no studies on the association between long-term cortisol levels (HCC) and dental anxiety. The FinnBrain Birth Cohort data provided an opportunity to study this association in pregnant women. Thus, the aim of this study was to examine the association between hair cortisol concentrations and dental anxiety at two points of pregnancy and to find out whether the association is stronger with anticipatory or treatment-related dental anxiety. Since HCC indicates retrospective cortisol secretion, we hypothesize that dental anxiety is associated with HCC levels, and that the association is stronger between HCC and anticipatory dental anxiety than with treatment-related dental anxiety, even when adjusted for covariates selected based on the literature.

## 2. Materials and Methods

This study is a secondary analysis of available data and uses a cross-sectional design.

*Study population.* The study population was drawn from the FinnBrain Birth Cohort study, the aim of which is to study genetic and environmental influence on child development and health outcomes. The study recruited pregnant women attending ultrasonography appointments in South-Western Hospital District, Finland and Åland Islands, Finland in 2011–2015. The total number of women recruited was 3808, 66% of those informed about the study [33]. The Intermunicipal Hospital District of Southwest Finland has given an ethical clearance for the FinnBrain Cohort Study in 14.6.2011 (14.6.2011 ETMK:57/180/2011 § 168). All participants gave signed informed consent for the study.

**Figure 1:**
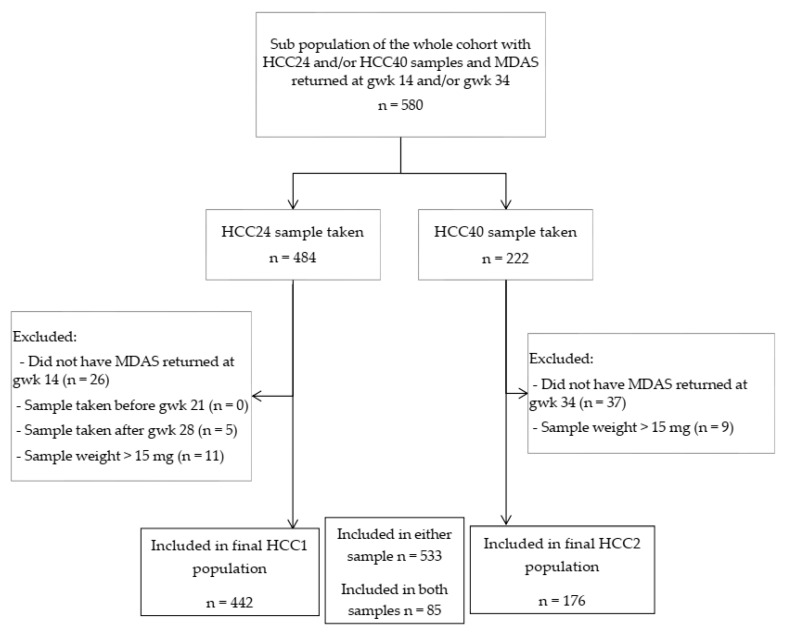
Flow chart of the study design. Abbreviations: gwk = gestational week, HCC24 = hair cortisol concentration measured at gwk 24, HCC40 = hair cortisol concentration measured at delivery, MDAS = Modified Dental Anxiety Scale.

The study population consisted of a sub-population of the cohort, including 533 women donating their hair samples at the gestational week (gwk) 24 and/or at delivery. Participants were divided into two groups representing the early and late stages of pregnancy and treated as separate groups. The first group consisted of women who donated hair samples at second-trimester study point at gestational week (gwk) 24 (HCC24 sample taken) and returned Modified Dental Anxiety Scale (MDAS) at gwk 14 (n = 442), hereafter redesigned as HCC1. The latter group donated hair samples at delivery (HCC40 sample taken) and returned MDAS at gestational week 34 (n = 176), hereafter redesigned as HCC2. (Figure 1) Of all subjects, 85 donated their hair and had MDAS returned at both study points and were therefore included in both groups.

*Hair cortisol concentration.* For the hair cortisol analysis, a strand of hair was cut from a standardized area of the posterior vertex region of the head as close to the scalp as possible. 5 cm segments of hair were analyzed to reflect cortisol secretion in the previous 5 months, based on estimated 1 cm hair growth per month [34]. The weight range of the analyzed samples was 5–15 mg. The exclusion of samples outside the weight range of 5–15 mg was based on sensitivity analyses suggesting that both very low and very high sample weights were related to increased sample weight-related HCC variance [32]. Hair samples were stored in foil in a dry place protected from light according to good research practice, Finnish legislation, and data protection until analyzed. The analyses were performed at the Life and Health Sciences Research Institute (ICVS), University of Minho, Portugal. The samples were processed according to a protocol previously described in detail [32] and cortisol concentration was analyzed with ELISA (IBL International Cortisol Saliva ELISA) following the manufacturer’s procedure. Samples were analyzed in duplicates and the mean coefficient of variation for HCC24 was 3.2% (SD 3.1). For HCC40, the mean coefficient of variation was 2.9% (SD 2.6).

Of the HCC24 samples taken before gwk 21 or after gwk 28 (n = 5) and/or samples weighing more than 15 mg were excluded (n = 11). Of the HCC40 samples weighing more than 15 mg were excluded as well (n = 9). The exclusion criteria were selected according to a study by Mustonen et al. [32] of the same cohort.

*Modified Dental Anxiety Scale.* Dental anxiety was measured with the Modified Dental Anxiety Scale (MDAS), a valid and widely used five-item instrument for self-rating dental fear [35,36,37]. MDAS has shown internal consistency (Crohnbach’s alpha = 0.93) and reliability over time (intraclass correlation coefficient = 0.93) [37].

The five items in the MDAS are: (1) if you went to your dentist for treatment tomorrow, how would you feel, (2) if you were sitting in the waiting room (waiting for treatment), how would you feel, (3) if you were about to have a tooth drilled, how would you feel, (4) if you were about to have your teeth scaled and polished, how would you feel, and (5) if you were about to have a local anesthetic injection in your gum, above an upper back tooth, how would you feel? Each item has five response options ranging from 1 (not anxious) to 5 (extremely anxious). The range for total sum score is 5–25. The cut-off point for high dental anxiety is established 19–25, representing high dental anxiety and 5–18 representing no to moderate dental anxiety [38]. The MDAS contains two separate factors: anticipatory dental anxiety (items 1 and 2, score range 2–10) and treatment-related dental anxiety (items 3, 4, and 5, score range 3–15) [21,39].

Both the dichotomized (high vs. no to moderate dental anxiety) and the continuous variable of the MDAS were used in this study. In addition, scores for the dental anxiety factors were calculated.

*Covariates.* Depressive symptoms, general anxiety symptoms, maternal age at delivery, maternal educational level, BMI before pregnancy, use of selective serotonin and/or noradrenalin reuptake inhibitors (SSRI/SNRI), and smoking during pregnancy were considered potential covariates based on prior literature [27,32,40,41].

To measure depressive and general anxiety symptoms, the participants were asked to fill in two self-report questionnaires, the Edinburgh Postnatal Depression Scale (EPDS) and anxiety subscale of the Symptom Checklist -90 (SCL-90). The EPDS was used to assess depressive symptoms the participants had been experiencing during the past 7 days. The EDPS is validated and consists of ten items scored from 0 to 3, the total score ranging from 0 to 30 [42,43]. The validated SCL-90 anxiety subscale was used to assess general anxiety symptoms experienced during the past month. The subscale consists of ten items scored from 0 to 4, the total score ranging from 0 to 40 [44,45]. The questionnaires were returned at the same time points as the MDAS, at gwk 14 in HCC1 group and at gwk 34 in HCC2 group.

Data on the other potential covariates were drawn from the Finnish Medical Birth Register (maternal age, BMI, and smoking status) and self-report questionnaires from gwk 14 or gwk 34 (educational level, medication) [33]. Educational level was categorized as low (high school/vocational), medium (polytechnics) and high (university or comparable).

*Statistical analysis.* All statistical analyses were performed using IBM SPSS^®^ Statistics version 25. Normality of continuous variables was checked using Q–Q plots and Shapiro–Wilk’s test. Normally distributed continuous variables are presented with means and standard deviations. The variables that are failing normality assumption are presented with medians and lower and upper quartiles. Natural logarithmic transformations were performed on HCC, MDAS sum scores, MDAS factor anticipatory dental anxiety, and MDAS factor treatment related dental anxiety, to meet the normality assumption.

The correlations between HCC and MDAS and its factors were analyzed with Pearson’s correlation using logarithmic transformed variables. A binary logistic regression model was fitted with the covariates to study the association between HCC and dental anxiety level (1 = high dental anxiety, 0 = no to moderate dental anxiety). Statistical significance was set as *p* < 0.05.

## 3. Results

Description of the study groups of pregnant women according to dental anxiety, HCC, and the potential covariates are presented in Table 1.

There were no statistically significant differences in depressive symptoms (*p* = 0.361), general anxiety symptoms (*p* = 0.214), maternal age at delivery (*p* = 0.998), BMI (*p* = 0.307), maternal educational level (*p* = 0.899), smoking status (*p* = 0.868) or the use of SSRI/SNRI (*p* = 0.150) between the two HCC groups.

The proportions of the two dental anxiety groups did not differ statistically significantly between HCC1 and HCC2 groups (high dental anxiety 5.2% vs. 5.1%, *p* = 0.86). Within the HCC groups, HCC was higher in the no to moderate dental anxiety group when compared to the high dental anxiety group (Table 2). The difference was statistically significant in the HCC1 group (*p* = 0.013), but not in the HCC2 group. The HCC median was significantly higher in the HCC2 group when compared to the HCC1 group (14.4 pg/mg vs. 10.8 pg/mg, *p* = 0.002).

Both groups showed very weak negative correlations, Pearson’s correlation coefficients from −0.099 to −0.052, between both HCC and MDAS sum score and HCC and MDAS factor scores (Table 3). Only anticipatory, but not treatment-related dental anxiety correlated statistically significantly with HCC in the HCC1 group (*p* = 0.039).

The binary logistic regression model showed that those participants with high dental anxiety had lower HCC levels when adjusted for depressive and general anxiety symptoms, BMI, smoking, and age in the HCC1 group, but not in the HCC2 group. (Table 4) In the HCC1 group, this means that for a 2SD decrease in HCC level, there was about 263% increased risk for having high dental anxiety, and for a 2SD increase in HCC level there was about 70% decreased risk for having high dental anxiety. In the HCC2 group the corresponding percentages were 160% and 62%, respectively, though the association was not statistically significant.

## 4. Discussion

In this study, we found that individuals with high dental anxiety had lower HCC levels when adjusted for depressive and general anxiety symptoms in the second trimester of the pregnancy, but the difference was not evident at the end of the pregnancy. Only anticipatory dental anxiety correlated with lower HCC levels during the second trimester of pregnancy.

This is, to our knowledge, the first study reporting on the association between long-term cortisol levels and dental anxiety. Our findings that HCC and dental anxiety were associated to the second trimester of pregnancy, but not at delivery, is aligned with other studies finding an association between prenatal psychological distress and HCC in mid-pregnancy [32,46,47,48], but not at the time of delivery [49,50]. The association sustained when adjusting for confounders. The fact that the association between HCC and dental anxiety was not statistically significant might be due to changes in cortisol levels and attenuated reactivity of HPA towards the end of pregnancy [24]. Decreased HCC levels in mothers experiencing high levels of dental anxiety is aligned with the results of previous studies of participants with generalized anxiety disorder and pregnancy-related anxiety and decreased HCC [32,51].

The cohort is very well characterized and the pipeline for collection of questionnaires and hair is systematic, ensuring the consistency of data collection. The biological sample sizes for HCC are relatively good compared to other studies during pregnancy [31]. The use of validated questionnaires is another strength.

However, the study also has limitations. The number of participants with high dental anxiety in the HCC2 group was small, which might be another reason for the not statistically significant association between HCC and dental anxiety. When comparing to the results on the entire population (n = 1984) from the same cohort, the prevalence of high dental anxiety and MDAS sums were slightly lower in both HCC groups (7% vs. 5%), whereas their anticipatory dental anxiety scores were slightly higher [23]. Prevalence of high dental anxiety was also lower in this study population than among a nationally representative sample of adult women of the same age (8% vs. 5%) in Finland [52]. These findings cannot be generalized to the non-pregnant population or other populations of different age or sex on which further studies are needed.

## 5. Conclusions

The independent association between HCC and dental anxiety suggests that long-term cortisol levels could play a role in the endogenous etiology of dental anxiety. As this is the first time such association is reported, and as the study was limited to pregnant women, replications in other populations are called for. In addition, whether this association is mediated via long-term stress or changes in HPA reactivity, needs further research.

## Figures and Tables

**Table 1:** Descriptive statistics of hair cortisol concentration measured at gwk 24 (HCC1) and at delivery (HCC2), MDAS and its factors, and potential covariates.

	**HCC1 (n = 442)**		**HCC2 (n = 176)**	
	Mean (SD)	Median (Q1–Q3)	Mean (SD)	Median (Q1–Q3)
Age at delivery	31.1 (4.3)		31.1 (4.2)	
BMI (kg/m2)	24.6 (4.5)	23.9 (21.4–26.7)	24.4 (4.7)	23.2 (21.2–26.2)
HCC (pg/mg)	19.2 (33.4)	10.8 (5.9–19.8)	22.4 (30.4)	14.4 (7.5–25.7)
Sample weight (mg)	10.0 (3.1)	9.9 (7.3–12.8)	11.1 (3.4)	11.8 (7.7–14.5)
MDAS sum	10.0 (4.1)	9.0 (7.0–12.0)	9.9 (4.1)	9.0 (7.0–11.0)
Anticipatory DA ^1^	3.4 (1.8)	3.0 (2.0–4.0)	3.2 (1.8)	3.0 (2.0–4.0)
Treatment related DA ^1^	6.6 (2.7)	6.0 (5.0–8.0)	6.6 (2.6)	6.0 (5.0–8.0)
SCL-90 score	3.2 (3.6)	2.0 (1.0–4.0)	3.1 (3.7)	2.0 (0.0–4.0)
EPDS score	5.0 (3.9)	4.0 (2.0–7.0)	4.7 (3.8)	4.0 (2.0–7.0)
	**HCC1 (n = 442)**		**HCC2 (n = 176)**	
	n (%)		n (%)	
**Educational level**				
High school/vocational	106 (24.0)		41 (23.3)	
Polytechnics	160 (36.2)		59 (33.5)	
University	175 (39.6)		71 (40.3)	
**Smoking**				
No	379 (85.7)		150 (85.2)	
Yes, before knowing about pregnancy	47 (10.6)		23 (13.1)	
Yes	16 (3.6)		3 (1.7)	
**Use of SSRI/SNRI**				
No	416 (94.1)		171 (97.2)	
Yes	19 (4.3)		3 (1.7)	

^1^ DA = dental anxiety.

**Table 2:** Descriptive statistics of hair cortisol concentrations (HCC) within the No to moderate and High dental anxiety (DA) groups at gwk 24 (HCC1) and at delivery (HCC2).

	HCC1 (n = 442)			HCC2 (n = 176)		
	n	Median (Q1–Q3)	*p*-Value ^1^	n	Median (Q1–Q3)	*p*-Value ^1^
No to moderate DA	419	11.3 (6.1–20.0)	**0.013**	167	14.7 (7.4–25.9)	0.534
High DA	23	6.2 (3.8–12.6)		9	11.9 (7.4–22.9)	

^1^ Wilcoxon rank sum test. Statistically significant *p*-values are bolded.

**Table 3:** Pearson correlation coefficients between dental anxiety (ln-transformed MDAS total score) and its factors, anticipatory dental anxiety and treatment-related dental anxiety, and ln-transformed HCC.

MDAS	HCC1 (n = 442)			HCC2 (n = 176)		
	r	*p*	n	r	*p*	n
Total score	−0.083	0.081	442	−0.067	0.377	176
Anticipatory dental anxiety	−0.099	**0.039**	441	−0.083	0.272	176
Treatment related dental anxiety	−0.069	0.148	442	−0.052	0.491	176

Statistically significant *p*-values are bolded.

**Table 4:** Final model presenting odds ratios (OR) with 95% confidence intervals (95% CI) and *p*-values on HCC for high dental anxiety adjusted for anxiety (SCL-90) and depressive (EPDS) symptoms, age, BMI, and smoking.

	HCC1 (n = 442)			HCC2 (n = 176)		
	OR	95% CI	*p*	OR	95% CI	*p*
HCC	0.548	0.35–0.86	**0.009**	0.611	0.28–1.33	0.216
SCL-90	0.894	0.74–1.08	0.237	0.827	0.62–1.11	0.204
EPDS	1.062	0.93–1.21	0.381	1.241	0.95–1.63	0.118
Age	0.968	0.88–1.07	0.516	0.912	0.75–1.10	0.341
BMI	1.112	1.03–1.20	**0.007**	0.971	0.83–1.13	0.699
Smoking	2.754	1.03–7.40	**0.045**	0.862	0.10–7.66	0.894

Statistically significant *p*-values are bolded.

## Data Availability

Data not available due to restrictions related to privacy and ethical issues.
